# Are Lean Body Mass and Fat-Free Mass the Same or Different Body Components? A Critical Perspective

**DOI:** 10.1016/j.advnut.2024.100335

**Published:** 2024-11-05

**Authors:** Steven B Heymsfield, Jasmine Brown, Sophia Ramirez, Carla M Prado, Grant M Tinsley, Maria Cristina Gonzalez

**Affiliations:** 1 Metabolism-Body Composition Core Laboratory, Pennington Biomedical Research Center, Louisiana State University System, Baton Rouge, LA, United States; 2 Department of Agricultural, Food & Nutritional Science, Human Nutrition Research Unit, University of Alberta, Edmonton, AB, Canada; 3 Department of Kinesiology and Sport Management, Texas Tech University, Lubbock, TX, United States; 4 Federal University of Pelotas, Post-graduate Program in Nutrition and Food, Pelotas, Brazil

**Keywords:** adiposity, body composition, obesity, malnutrition, muscle mass, fat-free mass, lean body mass

## Abstract

The 2-component molecular-level model dividing body mass into fat and fat-free mass (FFM) is a cornerstone of contemporary body composition research across multiple disciplines. Confusion prevails, however, as the term lean body mass (LBM) is frequently used interchangeably with FFM in scientific discourse. Are LBM and FFM the same or different body components? Captain Albert R. Behnke originated the LBM concept in 1942 and he argued that his “physiological” LBM component included “essential” fat or structural lipids whereas FFM is a chemical entity “free” of fat. Classical experimental animal and human studies conducted during Behnke’s era laid the foundation for the widely used body density and total body water 2-component molecular-level body composition models. Refined body composition models, organization of lipids into structural and functional groupings, and lipid extraction methods all have advanced since Behnke’s era. Our review provides an in-depth analysis of these developments with the aim of clarifying distinctions between the chemical composition of LBM and FFM. Our retrospective analysis reveals that FFM, derived experimentally as the difference between body weight and extracted neutral or nonpolar lipids (mainly triglycerides), includes polar or structural lipids (that is, Behnke’s “essential” fat). Accordingly, LBM as originally proposed by Behnke has the same chemical composition as FFM, thus answering a longstanding ambiguity in the body composition literature. Bringing body composition science into the modern era mandates the use of the chemically correct term FFM with the elimination of the duplicative term LBM that today has value primarily in a historical context. Avoiding the use of the term LBM additionally limits confusion surrounding similar widely used body composition terms such as lean mass, lean soft tissue mass, and lean muscle mass.

**Statement of Significance**

This study directly addresses a longstanding unresolved question if the 2 widely used body composition terms, “lean body mass” and “fat-free mass,” are the same or different body components. Our in-depth critical review established that lean body mass and fat-free mass are chemically identical body components, thus resolving a pervasive ambiguity that has led to past and current confusion in the scientific literature and public discourse on major topics in human nutrition.

## Introduction

The terms lean body mass (LBM) and fat-free mass (FFM) are often used interchangeably in human body composition literature, leading to confusion about their exact meanings and measurement methods. Are LBM and FFM the same or different body components? To answer this question, we first explore the origins of the term “LBM” and show that, at its inception, LBM reportedly differed from FFM only in including what was referred to as “essential fat” [1]. That distinction between LBM and FFM led us to then set out on a deep analysis of lipid terminology and classification, followed by a review of methods used to analyze tissue lipids. We show how advances in the study of lipid biology paralleled the development and refinement of body composition methods, models, and terminology. Lastly, we synthesize this collective information to advance a perspective on body composition terminology, specifically focusing on the question if LBM and FFM are the same or different body components.

## Lean Body Mass

### Concept origin

**FIGURE 1:**
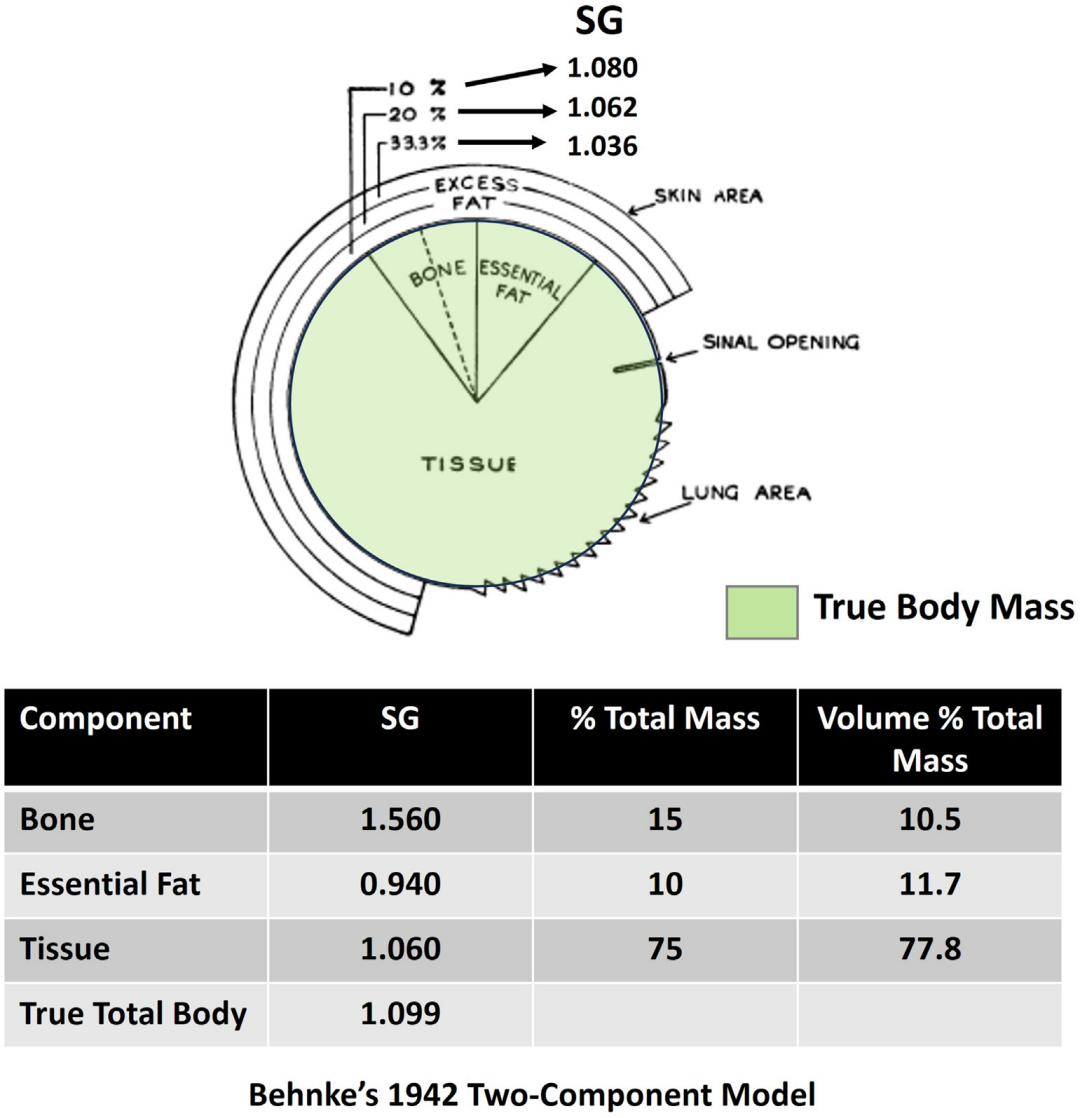
Behnke’s 1942 2-component body composition model consists of a “true” body mass core (green) with an encircling layer of “excess” fat. Behnke gives estimates of the 3-core component SG (bone, tissue, essential fat) and the impact on SG with addition of fat. Modified from Behnke, 1942 [1]. Behnke’s true body mass evolved into the component he termed “lean body mass.” SG, specific gravities.

It was 1942 and World War II was raging in Europe and the Pacific when Captain Albert R. Behnke Jr. delivered what was to become a classic Harvey Lecture and associated publication [1]. A United States Navy physician, Behnke was acutely aware of potentially fatal conditions such as the bends that arise with the release of inert gasses following exposure to high pressure. Behnke’s presentation recounted his “special interest” in the “absorption or elimination of gaseous nitrogen, which in turn have permitted estimates of fat content and composition of the body of the healthy, adult male.” Other than skinfold measurements, few technologies at the time were available for quantifying body fat [2]. To study the effects of abnormal pressure environments on an individual, Behnke reasoned that it would be useful to separate body mass into 2 components, an “essential” portion he referred to as the “true body” and the other excess fat that strongly influenced his physiological calculations. Evaluating the specific gravity of deep-sea divers, Behnke laid out his 1942 body composition model, as shown in Figure 1 [1]. Behnke’s model included a “true body mass” core surrounded by “excess fat.” Three “relatively stable” components comprised the true core, bone, tissue, and essential fat, each with an established specific gravity. Essential fat was defined by Behnke as including “myelin in the spinal cord, fat in bone marrow, lecithin, cholesterol, and other lipid or lipoid matter.” Later sections of this perspective contrast Behnke’s historical use of the term “essential” fat with modern nutritional definitions of “essential” lipids and the confusion these conflicting lipid definitions have introduced into the scientific literature. Behnke described the impact of adding varying amounts of excess body fat on specific gravity as shown in the figure. Two key inferences were made, that the specific gravity of “true” body mass is 1.099 and that of “excess” fat is 0.94. The proposed specific gravity of the true body (1.099) reflected the proportional contributions of bone, tissue, and essential fat. That same year, 1942, Behnke and his colleagues [3] introduced specific gravity, measured by underwater weighing, as an index of obesity. Behnke used the term specific gravity for his measurements, although specific gravity and density are not the same. A substance’s specific gravity is the ratio of its physical density to the density of water at a specified temperature and atmospheric pressure; specific gravity is unitless. Density (*ρ*) is the mass (*m*) per unit volume (*V*) of a substance (*ρ* = *m/V*). Body composition studies using underwater weighing methods now measure density as the temperature of the evaluated person and water are not always known [4].

### Concept refinement

**FIGURE 2:**
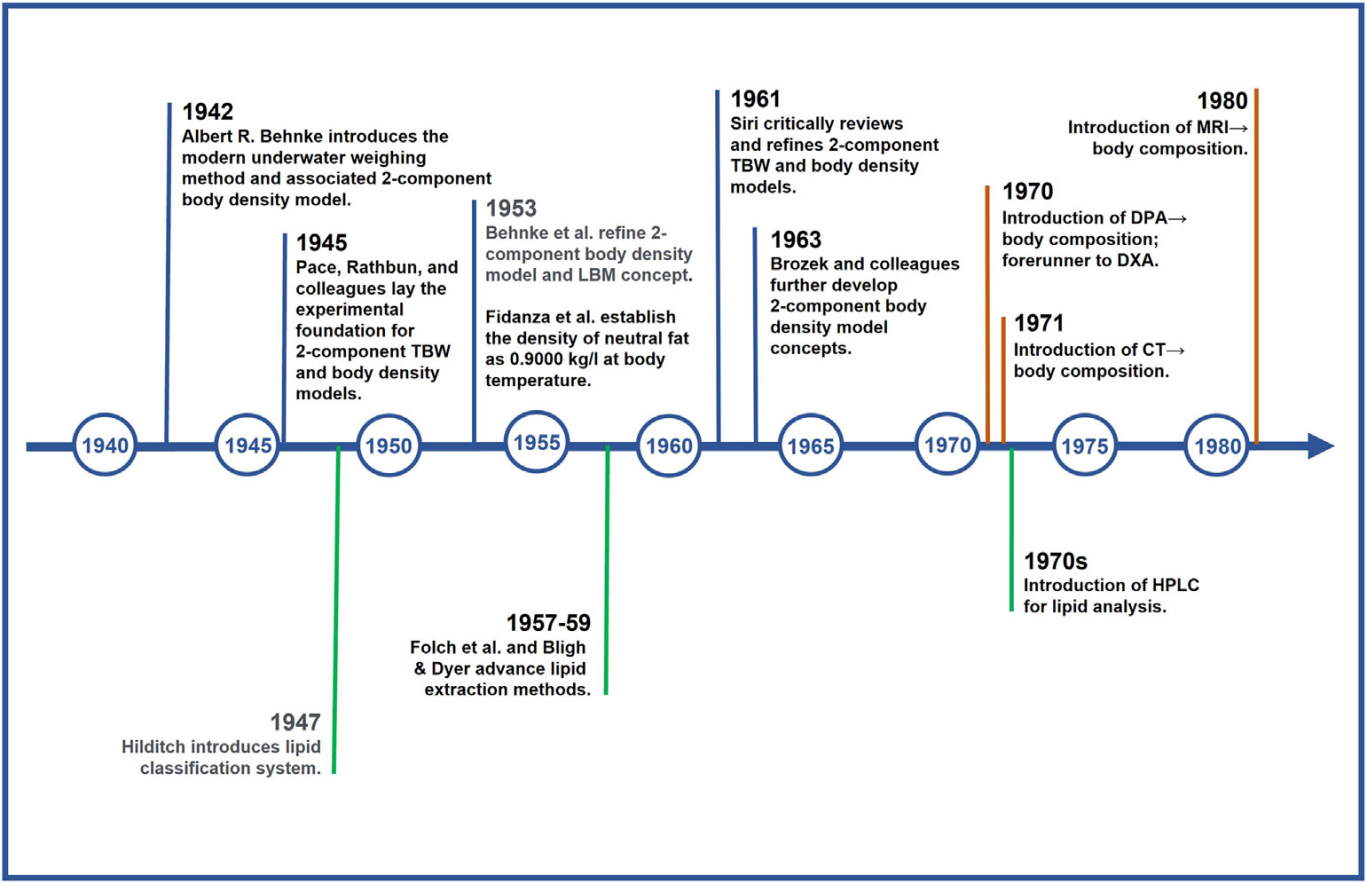
Timeline integrating salient advances in body composition and lipid analysis research up to 1980. Advances such as CT , DPA, DXA, and MRI (orange markers) led to new methods of evaluating body composition that were orthogonal to the 2-component body density method introduced by Behnke [1] in 1942 and by others that advanced his concepts (blue markers). Developments in lipid classification and analytics (green markers) began after Behnke’s 1942 report and continued after the 1980s with liquid chromatography-mass spectrometry techniques. Associated references are provided in the text. CT, computed tomography; DPA, dual-photon absorptiometry; DXA, dual energy X-ray absorptiometry; TBW, total body water.

**FIGURE 3:**
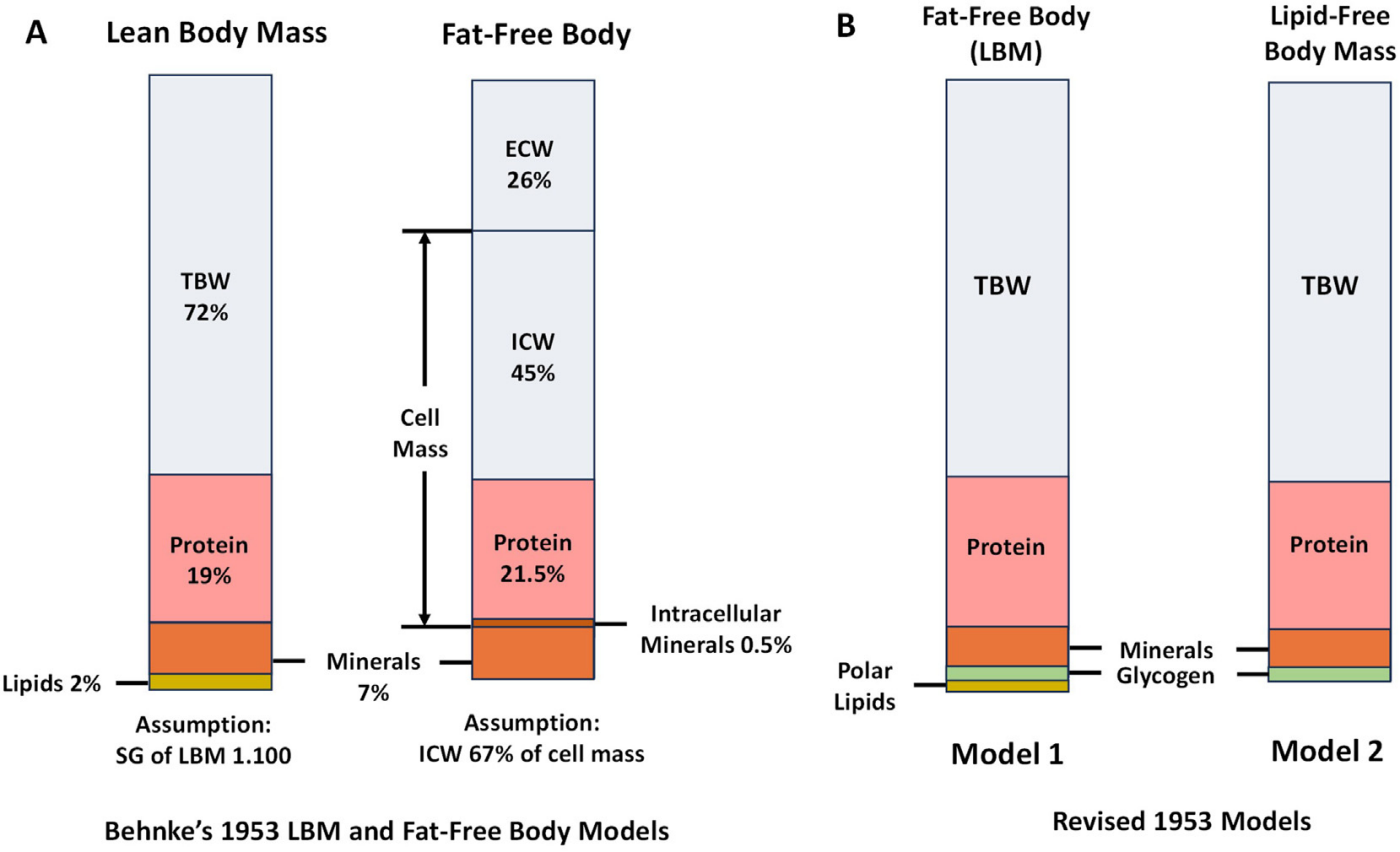
(A) Comparison of the LBM and fat-free body as adapted from Behnke, Osserman, and Welham’s 1953 publication [1]. **(**B) Reorganization of LBM and FFM models shown in (A) according to contemporary lipid terminology and extraction methods. ECW, extracellular water; FFM, fat-free mass; ICW, intracellular water; LBM, lean body mass; TBW, total body water.

Behnke and his colleagues published several more influential research papers [[5], [6], [7]] and book chapters [8,9] over the next several decades as the underwater weighing method became the reference approach for quantifying body fat [10], only supplanted by other emerging technologies in the late 1970s such as dual-photon absorptiometry, computed tomography, and magnetic resonance imaging (Figure 2) [1,2]. Behnke struck a common theme in all of these extensively documented publications. He viewed LBM, supported by growing evidence, as an “in vivo functional” entity in which the proportions of bone, water, and essential fat largely remain stable across people, including with interventions, and these 3 components collectively have a specific gravity of 1.100 [6,11]. In support of these observations, Behnke cited the classic 1945 studies of his colleagues at the Naval Research Institute showing that the percentage of “lean mass” as water is stable across mammals varying widely in size [12]. Behnke argued that even when all adipose tissue fat is depleted during starvation, the unquantified essential lipid (“perhaps 2–5% of LBM”) remains [8,13]. Behnke consistently made a sharp contrast to FFM, “an appropriate term for the chemically analyzed carcass free from fat”; that is, according to Behnke’s reasoning, FFM is devoid of essential fat as shown in a redrawn version of his published 1953 illustration (Figure 3A) [1,6]. In making this inference, Behnke reasoned that total body fat and lipid are chemically the same and that the 2 terms are synonymous, a conjecture we examine in detail later in this perspective.

Debate on the best underwater weighing model for estimating body fat continued for ≥2 decades following Behnke’s original publication with some of the most prominent scientists of that period expressing their diverging opinions. Most of the debate centered on if and how much “essential fat” was present in LBM. Several representative critical comments made by leading experts of that era are shown in Table 1 [8,14,15]. The authors of these comments came from their respective disciplines in chemistry and physiology with increasing understanding of the distinctions between “lipid” and “fat.” The current widely applied models relating body density and total body water (TBW) to body composition [16] evolved from these early discussions [17], although the terms LBM and FFM persist in our lexicon. In the following section, we untangle confusion stemming from the early use of body composition and lipid terminology with the ultimate aim of answering the question if LBM and FFM are the same or different body components.

**TABLE 1:** Quotes critical of Behnke’s LBM and “essential fat” concept by contemporaneous experts.

**Josef Brozek** [8] Brozek, editor of a series of body composition reviews, inserted a footnote in Behnke’s article that included the heading, Fat-Free and Lean Body Mass^1^ “^1^Editor’s Comment (J.B.): There has been an inordinate amount of confusion resulting from a loose usage of these terms. ‘Fat-free’ mass is clearly defined. It is the body weight less ‘total’ body fat, as determined by the specified technique, e.g., petroleum ether extraction.” “Lean body” mass is an ‘elastic’ concept, lacking precision, since—as Dr. Behnke points out—the amount of ‘essential’ lipid substances is not known at present and has been variously estimated, with values ranging from 10% to 2% of the fat-free weight.” **Flaminio Fidanza** [14] “Lean body mass was defined by Behnke in 1942 as the weight of the body less everything except ‘essential’ fat, and the latter was assumed to represent 10% of the lean body mass. Successively this percentage was reduced to 2%. Because the real amounts of essential fat present in cell membranes and of phospholipids in nerves and brain are difficult to assess in humans, the term “fat-free body mass” was preferred so as to avoid considerable confusion in the literature. At the joint FAO–WHO–UNU Expert Consultation on Energy and Protein Requirements held in Rome from 5 to 17 October 1981, with John Durnin and Gilbert Forbes, we finally decided to consider these 2 terms as synonymous and consequently lean body mass is now free of fat.” **Gilbert Forbes** [15] “The exact amounts of structural lipids, which are contained in cell membranes and as phospholipid in nerve and brain, present in the body have never been defined. It should be remembered that ‘structural’ lipids are not removed by the usual methods for fat extraction.”

### Sharpening nomenclature

Behnke first reported his original body composition model in 1942, long before contemporary model concepts and terminology were firmly in place. Today, we recognize 5 distinct body composition levels, atomic, molecular, cellular, tissue-organ, and whole-body, each with specific components and associated models [18]. Behnke’s original model published in 1942, shown in Figure 1, actually merges 2 body composition levels into LBM that includes components from the molecular (for example, “fat”) and tissue-organ levels (for example, bone). Quantitative development of contemporary body composition models separate components into their respective levels (for example, molecular-level model), something workers following Behnke recognized and later published in refined models and equations [19]. Behnke’s LBM model published 1 decade later in 1953, shown in Figure 3A, reflects improved taxonomy over his original 2-component model published in 1942; both LBM and fat-free body (that is, FFM) in his updated model are composed solely of molecular-level chemical components. These updated 2-component models distinguish LBM from FFM as differing only in “lipids,” the “essential fat” included by Behnke in LBM in his 1942 model shown in Figure 1. LBM and FFM models according to Behnke thus differed only in the presence of “essential” lipid in the 1953 LBM model. Exactly what constitutes “essential” lipid and body “fat”? Answering this question takes us down the path of exploring concepts in lipid biology that ultimately help us to disentangle the terms LBM and fat-free body that Behnke reasoned differed only in LBM including essential lipid and the fat-free body as lipid-free (Figure 3A).

## Lipid as a Body Composition Component

Total body lipid is a molecular-level component that comprises many different chemical species. At the outset, we provide a glossary (Table 2) [15,[20], [21], [22], [23], [24]] that has frequently used terms related to lipid biology in the following sections and a timeline integrating salient advances in lipid biology and body composition research are shown in Figure 2. Behnke’s foundational studies were conducted at a time when lipid biology was still in an early stage by comparison to contemporary biochemical concepts. Behnke introduced his 1942 model 5 y before the British chemist Thomas P. Hilditch published one the first lipid classification schemes, dividing simple lipids into greases and waxes (true waxes, sterols, and alcohols) [25]. Multiple classification schemes soon followed, organizing lipids according to composition (simple, complex, and derived), function (storage, structural, and as signals, cofactors, and pigments), and solubility (polar and nonpolar) [26,27]. Behnke used the terms fat and lipid interchangeably, although today “fat” is usually defined by chemists and regulatory agencies specifically as neutral (nonpolar) lipids, mainly triglycerides that serve as an endogenous source of metabolic energy [22,[28], [29], [30], [31]]. An even more narrow definition of fat is that it constitutes a family of triglycerides that are in solid form at room temperature. An unfortunate circumstance leading to confusion in the scientific literature is that the term “fat” is often used generically to describe all lipids and even adipose tissue, but in the discussions that follow we adhere to the aforementioned chemical definition of fat.

**TABLE 2:** Glossary of frequently used lipid terms.

**Total Lipids**: “A wide variety of natural products including fatty acids and their derivatives, steroids, terpenes, carotenoids and bile acids, which have in common a ready solubility in organic solvents such as diethyl ether, hexane, benzene, chloroform or methanol.” [20] **Polar Lipids**: “Polar lipids are amphiphilic lipids with a hydrophilic head and a hydrophobic tail. Polar lipids include phospholipids, sphingolipids, and glycolipids.” They are structural components of cell membranes and neural tissues. [15] **Nonpolar Lipids**: Nonpolar lipids are fatty molecules that have an even distribution of charge and are insoluble in water (a polar molecule) and only soluble in nonpolar solvents. Nonpolar lipids account for >90% of the total lipids with triglycerides the major type in humans. [21] **Fat**: In nutrition, biology, and chemistry, fat means any ester of fatty acids, or a mixture of such compounds. The term often refers specifically to triglycerides or even more narrowly to triglycerides that are solid or semisolid at room temperature. [22] **Neutral Lipids**: Nonpolar hydrophobic molecules that include triglycerides, cholesterol esters, and wax esters that provide organisms with energy. **Triglycerides**: Triglycerides (triacylglycerols) consist of 3 molecules of fatty acid combined with a molecule of the alcohol glycerol. Triglycerides are the most abundant lipid in human body. **Essential Lipids**: Two fatty acids (a-linolenic acid and linoleic acid) are required by humans for normal physiological function that cannot be synthesized in the body, either at all or in sufficient quantities, and must be obtained from a dietary source. [23] **Diethyl ether**: Nonpolar volatile organic solvent that consists of 1 oxygen atom linked to 2 ethyl groups. **Petroleum ether**: Petroleum ether is a petroleum distillation fraction consisting mainly of pentanes and hexanes that is commonly used as a nonpolar solvent for oils, fats, and waxes. [24] **Chloroform**: Volatile liquid (trichloromethane, CHCl_3_) frequently used as a nonpolar solvent. **Methanol**: Volatile alcohol (CH_3_OH) frequently used as a polar solvent.

Behnke’s fat-free body, as shown in Figure 3A, is thus really “lipid-free mass” by contemporary terminology. Behnke also used the terms “essential fat” and “essential lipid,” although today we only recognize 2 “essential” lipids that cannot be synthesized by the human body, the polyunsaturated fatty acids linoleic acid and α-linolenic acid [32]. Behnke was actually referring to essential fats or lipids as the “structural,” largely polar, lipids such as phospholipids that are present in cell membranes and neural tissues. These component taxonomy vagaries are partially responsible for the confusion surrounding the use of the terms LBM and FFM. We can now recast Behnke’s LBM and fat-free body molecular-level models in contemporary terms as shown here and in Figure 3B:

Model1:BodyWeight=FatMass(neutrallipids)+[BodyWeight-FatMass],

LBM=BodyWeight−FatMass

Model2:BodyWeight=TotalLipidMass+[BodyWeight−TotalLipidMass]

Lipid−FreeBodyMass=BodyWeight−TotalLipidMass

Model 1 is what Behnke described in 1953 as including structural, largely polar, lipids in the body mass remaining after extraction of body fat, which is “LBM.” Model 2 is what Behnke described as the “fat-free body” devoid of any lipid. Behnke’s observations in 1953 set the stage for posing the key question examined in this review: are “LBM” and “FFM” as currently measured in research laboratories and clinical settings the same or different components as defined by Behnke’s 2 models? Specifically, how were lipids allocated in classical body composition studies that form the basis of 2-component models? That is, were lipid extraction methods used that removed total body lipid from tissues (Model 2) or only neutral lipids (Model 1)? This question is relevant as these early studies set in place key “constants” that are used today in 2-component molecular-level body composition models based on body density and TBW [16]. Moreover, answering this question will tell us if LBM and FFM are indeed the same or different components. To examine this question, we first need to understand how lipids are extracted from and then measured in animal and human tissues. From there we can review the major experimental studies laying the groundwork for 2-component molecular-level models.

### Analysis methods

The first step in analyzing an animal or human’s whole-body or isolated tissue lipids is to carefully prepare the sample to avoid loss of compounds sensitive to oxidation, enzymatic hydrolysis, and other side reactions [20]. Other mechanical, chemical, and enzymatic pre-treatments may be required before sample desiccation and solvent extraction depending on the nature of evaluated tissues [33]. Covalently bound lipids may require a hydrolysis step for full extraction. Lipid extraction from the prepared sample is then typically accomplished using a solvent system that include polar and/or nonpolar solvents [33,34]. Polar lipids include molecules such as phospholipids, glycolipids, polysaccharides, and lipoproteins. These molecular species, which are both lipophilic and hydrophilic (that is, amphipathic), are involved in a variety of biological functions and are found mainly in cell membranes and neural tissues; as noted, polar lipids are likely what Behnke viewed as “structural” or “essential” fats [1]. Nonpolar or “neutral” lipids include acylglycerols, free-fatty acids, sterols, sterol esters, and waxes that are synthesized within cells as a form of storage energy, the main type triglycerides. As noted earlier, neutral, or nonpolar lipids constitute the “fat” present in humans and are mainly in the form of triglycerides.

The solvent systems in use today for extracting total tissue lipids emerged primarily from the methods reported by Folch et al. [35] in 1957 followed several years later in 1959 by Bligh and Dyer [36] (Figure 2). These methods, reported more than a decade after Behnke’s original publication [1], were based on a ternary system with 2 main solvents, nonpolar chloroform and polar methanol. Nonpolar and polar lipids can be separated using these methods with contemporary refinements [33,34]. Another approach, widely used in the meat production industry, is to extract “crude” fat with analytical protocols [26,37] including diethyl ether (“ether”) or petroleum ether. Petroleum ether is a highly flammable nonpolar aliphatic hydrocarbon solvent mainly consisting of hexanes and pentanes that is more hydrophobic than diethyl ether and thus has greater selectivity for more hydrophobic lipids [20].

Several studies have compared Folch and Bligh and Dyer with ether and petroleum ether lipid extractions of plant and biological samples. Most studies report greater amounts of lipid extracted with Folch/Bligh–Dyer than with ether extractions, the between-method difference primarily polar lipids (Table 3) [[38], [39], [40], [41], [42]]. That is, Folch/Bligh–Dyer extracts “total” lipids whereas ether extracts mainly “fat” or neutral lipids. Although we recognize there are many nuances to these assertions, this construct is useful in understanding the experimental studies that serve as the foundation of 2-component body composition models. The key question is which model (Mode 1 or Model 2), or both, is supported by experimental studies that formed the basis of contemporary 2-component body composition methods?

**TABLE 3:** Comparison of representative lipid extraction studies using petroleum ether and chloroform-methanol (Folch method) as solvents.

**First author** (year; reference)	**Tissue**	**Ether**	**Folch**	**Observation**
**Hubbard** (1977; [38])	Various food products	AOAC (petroleum ether)	Chloroform-methanol (2:1vol/vol)	Largest amount of total fat, sterols, and cholesterol extracted with the Folch method.
**Rhee** (1988; [39])	Beef longissimus muscle	AOAC (Petroleum ether)	Chloroform-methanol (2:1vol/vol)	Fat values extracted from raw samples were 1.062-fold higher with the Folch method.
**Pérez-Palacios** (2008; [40])	Various muscle meat products	ST-SOX (Petroleum ether)	Chloroform-methanol (2:1vol/vol)	The largest amount of lipids extracted with the Folch method.
**Dow** (2010; [41])	Beef longissimus muscle	AOAC (Petroleum ether)	Chloroform-methanol (2:1vol/vol)	More fat extracted (6.9%) with the Folch method.
**Fiori** (2013; [42])	Various lamb muscles	AOAC (Petroleum ether)	Chloroform-methanol (2:1vol/vol)	Larger amount of lipid extracted with Folch method compared with petroleum ether.

Abbreviations: AOAC, Association of Analytical Chemists; ST-SOX, standard Soxhlet method.

### Experimental studies

Several years following Behnke’s original body composition publication, Pace, Rathbun, Smith, and Morales published a series of 3 milestone papers in 1945 [12,43,44]. The studies by this group laid the foundation for the widely used the TBW method for measuring total body fat that relies on the assumed constant TBW/FFM ratio of 0.732 [44] (Supplemental Figure 1). The first paper in the series described chemical analyses of eviscerated guinea pig carcasses that included skin and skeleton [44]; fat extraction was with petroleum ether. The investigators thus extracted primarily neutral (nonpolar) lipids (that is, fat); we can conjecture that most polar lipids were left behind during the extraction process, thus favoring Model 1 that includes lipids in the residual mass after extraction of body fat. The classic model constant TBW/FFM = 0.732 is thus based on FFM, as derived in Model 1.

A second pivotal study was conducted by a prominent group of scientists working at the University of Minnesota’s Laboratory of Physiological Hygiene led by Ancel Keys [45]. The underwater weighing method introduced by Behnke includes a key constant, that of “fat” density, as part of the 2-component body density model. Fidanza, Keys, and Anderson from the Minnesota group published an influential paper reporting the density of fat in man and other mammals [4]. Fidanza and his colleagues measured the density of fat extracted from subcutaneous and internal adipose tissue biopsies in 5 adults and arrived at the mean value of 0.9000 g/cc at 37°C. Adipose tissue samples in this study were extracted with diethyl ether and petroleum ether, and thus, the evaluated “fat” consisted mainly of neutral lipids or triglycerides. Newman et al. [46] reported an almost identical study to the one reported by Fidanza et al. [4] in 1972 comparing Folch to Skellysolve B extraction of human adipose tissue samples; Skellysolve B is a nonpolar solvent similar to petroleum ether. Triglycerides were similarly extracted from adipose tissue by both protocols (Folch/Skellysolve B, 782 compared with 723 mg/g) whereas lesser amounts of phospholipid were extracted with Skellysolve B (22.7 compared with 1.12 mg/g). As currently applied, the 2-component body density model with the density of fat set at 0.9000 g/cc thus conforms to Model 1; the evaluated density of tissue extracts was for “fat” and not “lipid”.

Rathbun et al. and Keys et al. reviewed other contemporary experimental animal and human cadaver studies in addition to their own seminal contributions. Keys and Brozek [47] and later Brozek et al. [11] used data from these experimental studies to further refine the 2-component body density model with confirmation of FFM density at 1.100 g/cc. The lipid extraction methods of these studies are summarized in Table 4 [4,11,44,[48], [49], [50], [51], [52], [53], [54]], and most of them are based on ether or petroleum ether as main solvents. Although we cannot know exactly what lipids were extracted in these studies, our retrospective analysis favors Model 1 (that is, on the basis of the use of nonpolar extraction solvents) as the model that was investigated in most of these early body composition studies. If we apply current lipid definitions, FFM and Behnke’s 1953 LBM are thus the same components and align with Model 1 as shown updated with contemporary terminology in Figure 3B.

**TABLE 4:** Tissue lipid extraction methods cited in classic animal and human body composition studies.

**First author** (year; reference)	**Source**	**Preparation**	**Extraction method**
**Animal Studies**^1^			
**Hatai** (1917; [48])	Albino rat	Eviscerated carcass; dried and homogenized tissue	Extraction with alcohol and ether.
**Light** (1934; [49])	Rat	Eviscerated carcass	Dried tissue boiled in anhydrous alcohol-ether mixture; residue extracted with petroleum ether.
**Harrison** (1936; [50])	Rabbit, dog, and monkey	Whole carcass and various organs and tissues	Ground and dried material, first partial fat extraction with petroleum ether and then with alcohol and ether.
**Ashworth** (1938; [51])	Albino rat	Dried carcass	Dried material extracted with ether.
**Rathbun** (1945; [44])	Guinea pig	Eviscerated carcass; dried and homogenized tissue	Soxhlet^3^ extraction with petroleum ether.
**Human Studies**			
**Mitchell**^2^ (1945; [52])	Human cadaver	Isolation and analysis of individual organs and tissues	Ether extract according to the AOAC method.
**Widdowson**^2^ (1951; [53])	Human cadaver	Isolation and analysis of individual organs and tissues	Acid hydrolysate washed with alcohol.^4^
**Forbes**^2^ (1953; [54])	Human cadaver	Soft tissues	Soxhlet^3^ extraction with petroleum ether.
**Fidanza** (1953; [4])	Human biopsy samples Other species: dog, rat, rabbit, Guinea pig, steer, pig, and lamb	Subcutaneous and internal surgical samples Subcutaneous and internal samples	Soxhlet^3^ extraction with ether and petroleum ether. Extraction with ether and petroleum ether.

Abbreviation: AOAC, Association of Analytical Chemists.

1 Cited by Rathbun et al. [44] in 1945 as shown in Supplemental Figure 1.

2 Cited by Brozek and Keys [11] in 1963.

3 Soxhlet extraction is a widely used continuous solvent cycling process.

4 The authors cite several previous studies for the fat extraction method, none of which fully clarify the chemical procedures.

A critical feature of 2-component molecular-level models is the stability of applied “constants” such as TBW/FFM (0.732) and the density of FFM (1.100 g/cc) [55]. One concern with Behnke’s LBM model was that “fat” is labile and changes in relative amount with variation in energy balance. If lipids are present in LBM and a person lost weight, then these lipids might be catabolized, thus potentially changing the density of FFM. Behnke’s argued, by citing literature, that “essential” fat was present even in people succumbing with starvation [13]; like TBW, he postulated that “essential” fat is a relatively stable proportion of “LBM.” This hypothesis was tested by Comizio et al. [56] who evaluated the effects of weight loss and exercise on total body and nonpolar lipids (triglycerides) in adult rats. The rats were divided into 3 groups: baseline control; 9-wk calorie-restricted; and 9-wk calorie-restricted plus exercise. Total lipid was extracted from carcass homogenates using the method of Bligh and Dyer [36]; homogenates were also analyzed for triglyceride (neutral lipid) using a colorimetric assay. We thus have the lipid analyses captured with Models 1 and 2. Total body lipid and triglyceride at baseline were 17.1% and 14.3% of body weight, respectively. The calculated nontriglyceride lipid, consistent with Behnke’s “essential fat” [1], was 2.8% of body weight. As expected, calorie restriction without or with exercise led to triglyceride mobilization from adipose tissue with a reduction in the proportion of total lipid as triglyceride from baseline at 83%–70% and 67% at the 9-wk body composition evaluation; nontriglyceride lipid as a proportion of body weight remained relatively unchanged from baseline (2.8%) at 2.7% and 2.2% (*p* = NS), respectively. We can derive the percentage of triglyceride-free mass (that is, FFM) as remaining lipids from the mean values published by Comizio et al. [56] as 3.2% at baseline and with calorie restriction without and with exercise as 2.9% and 2.2%, respectively. Thus, the polar (“essential”) lipids according to these estimates are roughly 2% and 3% of triglyceride-free mass and appear to be stable with respect to substantial changes in weight and adiposity. Future experimental studies are needed to critically evaluate structural/polar lipids as a body composition component under varying dietary and activity conditions.

## Summary and Perspective

Captain Albert R. Behnke’s introduction of the 2-component underwater weighing method for estimating body fat in 1942 is 1 of the milestones in body composition research [1]. His approach, 8 decades later and with refinements, is still in use today [55]. One legacy of Behnke’s method is the persistence of the term LBM in our current body composition vocabulary even though its exact meaning in terms of chemical components remains ambiguous. Here, we traced through how concepts in body composition modeling and lipid science evolved over time since Behnke’s introduction of the term LBM and how this evolution has distorted our ability to critically evaluate exactly what “LBM” is with respect to modern terminology. First, the development of body composition models became more rigorous as the field gained acceptance as a formal scientific discipline [2,10,18]. Behnke’s use of LBM component terms such as “tissue” or “bone” in 1942 [1] gave way to molecular-level components such as protein, water, and bone minerals 1 decade later [6] as new investigators with rigorous training in chemistry and physiology entered the field [8,11,17,47]. Tensions grew as Behnke adhered to the view that LBM was a “living” functional construct whereas FFM was a chemically defined entity developed from the in vitro analysis of animal and human tissues [6]. Uncertainty arose on exactly what was meant by “essential fat” and how this relatively small lipid mass is allocated in 2-component molecular-level body composition models (Table 1).

The second development was the evolution of lipid science with recognition of myriad types of lipids and multiple classification schemes that followed [26,29,20,33,34]. Terms such as “fat” took on specific meaning from the chemical perspective [22,[28], [29], [30], [31]], a refinement from the loose use characteristic of Behnke’s era and that exists in common jargon today. Behnke’s “essential fat” reflects this ambiguity as today we define only 2 specific “essential” lipids [32]. The third development also followed in lipid science with improving methods for separation of individual lipids and groups of lipids. Today, there are powerful methods of isolating and then identifying individual lipid types present in animal and human tissues [20,26,29,33,34]. In the decades leading up to and during Behnke’s era, there was a clear recognition that “fats” are soluble in solvents such as diethyl ether. Most of the studies relating to body composition, both animal and human, involved variations in ether extraction methods as summarized in TABLE 2, TABLE 3. Diethyl ether and petroleum ether are now recognized largely as nonpolar lipid solvents [20,26,29,33,34]; thus, ether extractions of tissues include mainly neutral lipids consisting primarily of triglycerides, or in modern parlance, “fat” [22,[28], [29], [30], [31]]. However, the specific details of extraction methods are important, and we cannot be certain of exactly what lipids were removed from tissues during early body composition studies, nor can we be sure of what lipids remained behind in the “fat-free body.” By conjecture, we can assume that some polar lipids, Behnke’s “essential fat,” were probably included in the measured “LBM.” From this ambiguity grew our foundation for 2-component molecular-level models based on body density and TBW [12,17]. Using contemporary terminology brought us to the conclusion that LBM and FFM are the same in terms of their chemical makeup. That is, both LBM and FFM include what might be referred to in Behnke’s terms as “structural” lipids. We can surmise that Behnke’s models drawn in Figure 3B were labeled incorrectly when viewed from our contemporary perspective; as shown in Figure 3B, LBM and fat-free body are chemically the same whereas the original fat-free body is actually lipid-free mass. A critical feature of LBM and FFM recognized by investigators over the decades is the concept that they are relatively stable in chemical makeup, even with fluctuations in energy balance and variation in age and race/ethnicity [5,6,16,17,19]. Although some deviations from this concept of FFM chemical stability are recognized [57], most modern studies report values for FFM hydration and density at or very close to their assumed constant respective values of 0.732 and 1.100 g/cc.

This overview brings us to the critical recognition that Behnke’s “essential fat,” specifically structural or polar lipids, must be present in one or the other body components (that is, fat or FFM), but beyond our review here their measurement is missing in critical body composition studies. If allocated to the fat component, then we are considering total lipid mass, and the associated density will not be exactly 0.9000 g/cc. Similarly, if non-“fat” lipids are allocated to FFM, they have not been factored into the density of FFM at 1.100 g/cc [11,17,47]. We leave this observation for future discussions and critical analyses, and at this point, we do not favor any revisions in body density and TBW 2-component models [55]. An important recognition in this context is that current 2-component molecular-level TBW and body density models for estimating total body fat do not consider components in very small amounts such as nucleic acids, often largely ignore air or fecal matter within the gastrointestinal tract, and cannot accurately incorporate amounts of rapidly changing compounds such as glycogen. An intangible error thus surrounds current body composition estimates based on TBW and body density models, although both approaches currently give total body fat estimates that are in close agreement [14]. However, the preponderance of evidence available now suggests that LBM and FFM are the same body components and chemically correct language favors the use of the term FFM *and elimination of LBM in discussions involving body composition*. Endless confusion reigns in publications and presentations that loosely use LBM, FFM, and even “lean mass,” “lean soft tissue mass,” and “lean muscle mass” as the same body components even though actual measured components may be different, and we outline some of these concerns in Table 5. The table includes our proposed component definitions consistent with contemporary body composition terminology.

**TABLE 5:** Proposed definitions of relevant body composition components consistent with current measurement methods and terminology.

Term(s)	Proposed Definition	Level	Recommendations
FFM LBM	The estimated mass of all nonfat molecules in the body, regardless of where they occur. In this case, ‘fat’ refers to nonpolar lipids, mainly triglycerides.	Molecular	To avoid confusion and improve scientific rigor, FFM is the preferred term for this body component, which is often measured with techniques using 2-compartment models such as TBW, body density, BIA, DXA, and 3D optical imaging. The term LBM should be reserved for historical discussions of body composition methods.
LM	The term “lean mass” generally refers to nonfat or non-AT components.	Not specified	Lean mass should only be used in discussions as a synonym of FFM and adipose tissue free mass but not as an alternative to DXA-measured lean soft tissue mass. The term lean mass should only be used when referring to all lean components, including bone mineral.
LST	The estimated mass of all nonfat, nonbone mineral molecules in the body, regardless of where they occur. In this case, ‘fat’ refers to nonpolar lipids, mainly triglycerides.	Molecular	This term should only be referred to when using techniques that can accurately estimate this component such as DXA, or using appropriately validated prediction equations based on such techniques. The term LST should be reported instead of LM to specify the noninclusion of bone mineral. LST should not be confused with skeletal muscle mass as the 2 are different components. Appendicular LST includes the LST in all 4 extremities.
SMM	The estimated mass of all anatomically defined skeletal muscles in the body.	Organ/Tissue	This term should only be referred to when using techniques that are actually estimating skeletal muscle tissue such as MRI, or using appropriately validated equations based on such techniques. As such, SMM is the muscle tissue attached to bones and is responsible for movement; by contrast, LST includes all nonfat and nonbone mineral components, including SMM, nonfat components of adipose tissue, skin, connective tissue, etc. Appendicular LST should not be confused with appendicular SMM.
LMM	LM in an amalgam of the terms “lean body mass” and “skeletal muscle mass,” which is unclear in its true meaning and has limited usefulness.	Unclear	The use of this term is discouraged. Rather, “FFM” or “skeletal muscle mass” should be used, as appropriate.

Abbreviations used: BIA, bioimpedance analysis; DXA, dual energy X-ray absorptiometry; FFM, fat-free mass; LBM, lean body mass; LM, lean mass; LMM, lean muscle mass; LST, lean soft tissue; SMM, skeletal muscle mass.

Our overall suggested approach to defining and using rigorous terminology in the scientific literature and public discourse will lend clarity and rigor to our field. A summary of the main highlights of our review is shown in Table 6.

**TABLE 6:** Review highlights.

• **Underwater Weighing Method:** ○ Introduced by Behnke [1] in 1942, this approach for estimating total body fat remains in use today, sometimes with technological advances [55]. Behnke established the 2-component body density model that divides body mass into “fat” and “LBM”. • **Legacy of LBM Term:** ○ The term LBM is still used by investigators in presentations and throughout scientific literature but lacks a clear definition in modern chemical terms. • **Evolution of Body Composition Models:** ○ Molecular-level models have become more rigorous, moving from general terms like “tissue” to chemical components such as protein and water. Fat is 1 type of chemical component, a lipid category that’s mainly in the form of triglycerides. Nonfat lipids are primarily polar molecules that have many structural and metabolic functions. Behnke posited that LBM included “essential lipids” whereas “FFM” was devoid of all lipids. Today only 2 “essential” lipids are known to be required in the diet, Behnke’s “essential” lipids now recognized as the “nonfat” lipids extracted from tissues with polar solvents. • **Two-Component Models:** ○ Ambiguities in early lipid extraction methods led to confusion surrounding current models based on body density and TBW. LBM and FFM are chemically similar according to experimental studies, both including multifunction nonfat lipids. The body “fat” (that is, many triglycerides) component, according to this concept, serves as a source of metabolic energy. At present, there are no published lipid-free molecular-level 2 component models. • **Chemical Stability:** ○ LBM and FFM are considered chemically stable by investigators, with some recognized deviations. The nonfat component of FFM, a relatively small mass, is largely un-studied and can form the basis of future body composition investigations. • **Which term to use?** ○ Because LBM and FFM are chemically the same (that is, both include nonfat lipids), the term LBM should be replaced in presentations and scientific reports with FFM (that is, a term with chemically correct taxonomy) to avoid confusion and improve scientific rigor in body composition studies.

Abbreviations: FFM, fat-free mass; LBM, lean body mass.

## Author contributions

The authors’ responsibilities were as follows – SBH, JB, SR, CP, GMT, MCG: designed the review outline; SBH, JB, SR, CP, GMT, MCG: provided essential materials; SBH, JB, SR, CP, GMT, MCG: analyzed data; SBH, JB, SR, CP, MCG, GMT: wrote the paper; and all authors read and approved the final version.

## Funding

This work was partially supported by National Institutes of Health NORC Center Grants P30DK072476, Pennington/Louisiana, P30DK040561, Harvard. CMP is partially funded through the Canada Research Chairs Program.

## Conflict of interest

SBH serves on the Medical Advisory Boards of Tanita Corporation, Novo Nordisk, Abbott, Novartis, Versanis, and Medifast. GMT has received support for his research laboratory, in the form of research grants or equipment loan or donation, from manufacturers of body composition assessment devices, including Size Stream LLC; Naked Labs Inc.; Prism Labs Inc.; RJL Systems; MuscleSound; and Biospace, Inc. None of these entities played a role in the present work.
