# Supplementary material for: Are Lean Body Mass and Fat-Free Mass the Same or Different Body Components? A Critical Perspective
Source: Adv Nutr. 2024 Nov 5;15(12):100335. doi: 10.1016/j.advnut.2024.100335 (PMC11625996; doi:10.1016/j.advnut.2024.100335)
Supplement: Multimedia component 1 [file mmc1.docx]

**SUPPLEMENTARY MATERIAL**

**Are Lean Body Mass and Fat-free Mass the Same or Different Body Components? A Critical Perspective**

Steven B. Heymsfield^1^, Jasmine Brown^1^, Sophia Ramirez^1^, Carla M. Prado^2^, Grant M. Tinsley^3^, Maria Cristina Gonzalez^4^

^1^Pennington Biomedical Research Center, Louisiana State University System, Baton Rouge, LA, USA; ^2^Department of Agricultural, Food & Nutritional Science, Human Nutrition Research Unit, University of Alberta, Edmonton, Alberta, Canada; ^3^Department of Kinesiology and Sport Management, Texas Tech University, Lubbock, TX, USA; ^4^Federal University of Pelotas, Post-graduate Program in Nutrition and Food, Pelotas, Brazil.

**Address Correspondence to**:

Steven B. Heymsfield, M.D.
Pennington Biomedical Research Center
6400 Perkins Road
Baton Rouge, LA 70808

**Tel**: 225-763-2541

**Fax**: 225-763-3030

**E-mail**: [Steven.Heymsfield@pbrc.edu](mailto:Steven.Heymsfield@pbrc.edu)

**SUPPLEMENTARY FIGURE 1


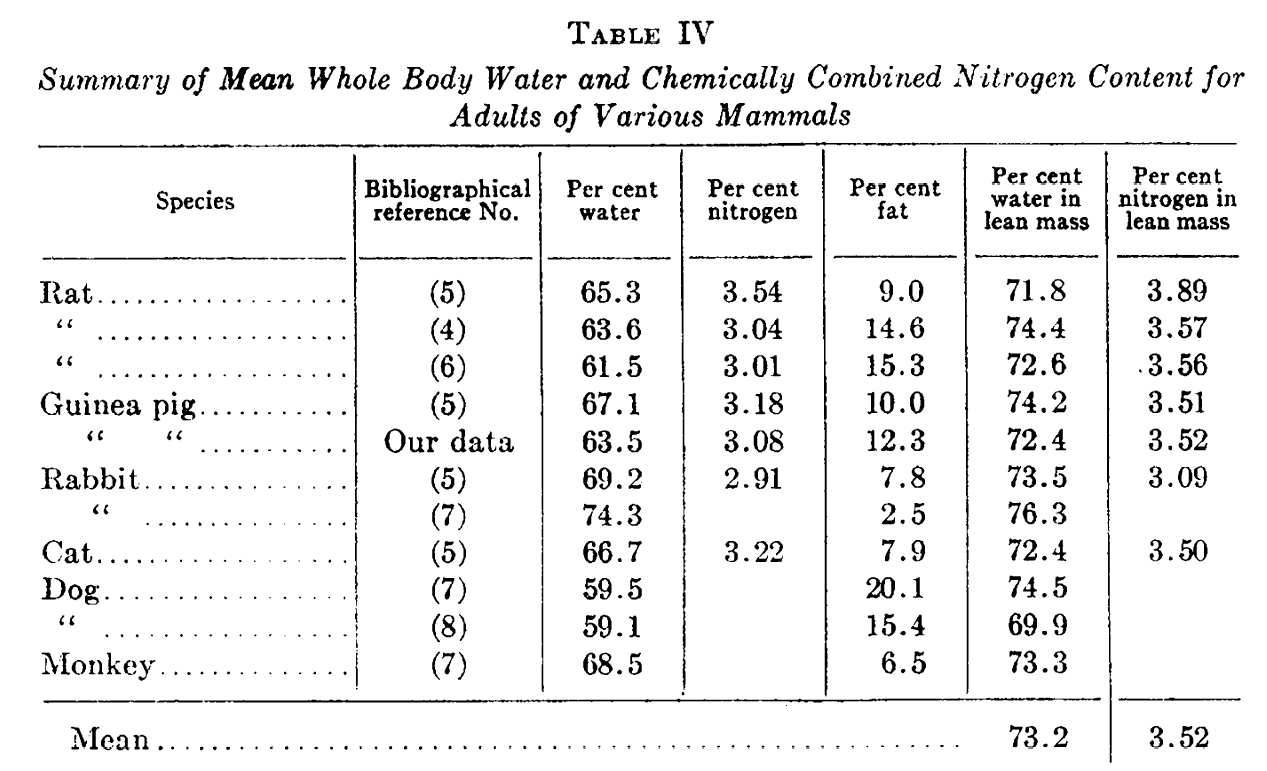
**

Table from Pace and Rathbun’s classic paper (1) summarizing body composition studies in animals showing the stable hydration of lean mass, defined as the difference between body mass and ether-extracted “fat”. Cited publications are presented in Table 4 of the manuscript. With permission from the publisher. Reference 8 was from Behnke (2) and did not involve a lipid extraction method. “Our data” was from Pace (1). Remaining references (4-7) are cited below.

1. Pace N, Rathbun EN. Studies on body composition: III. The body water and chemically combined nitrogen content in relation to fat content. J Biol Chem. 1945;158:685-91.
2. Behnke AR. Physiologic Studies Pertaining to Deep Sea Diving and Aviation, Especially in Relation to the Fat Content and Composition of the Body: The Harvey Lecture, March 19, 1942. Bull N Y Acad. Med. 1942;18:561-85.

4. Ashworth US, Cowgill GR. Body composition as factor governing basal heat production and endogenous
 nitrogen excretion. J Nutr. 1938;115:73-81.

5. Hatai S. Changes in the composition of the entire body of the albino rat during the life span. American
 Journal of Anatomy. 1917;21:23-37.

6. Light AE, Smith PK, Smith AH, Anderson WE. Inorganic salts in nutrition. XI. Changes in composition
 of the whole animal induced by a diet poor in salts. J Biol Chem. 1934;107:689-95.

7. Harrison HE, Darrow DC, Yannet H. The total electrolyte content of animals and its probable relation to
 the distribution of body water. J Biol Chem. 1936;113:515-29.
